# Strong association of lumbar disk herniation with diabetes mellitus: a 12-year nationwide retrospective cohort study

**DOI:** 10.3389/fendo.2023.1260566

**Published:** 2023-11-02

**Authors:** Jing-Xing Li, Tzu-Ju Hsu, Shu-Bai Hsu, Yu-Hsiang Lin

**Affiliations:** ^1^ Department of Internal Medicine, Taipei Veterans General Hospital, Taipei, Taiwan; ^2^ School of Medicine, China Medical University, Taichung, Taiwan; ^3^ Graduate Institute of Clinical Laboratory Sciences and Medical Biotechnology, National Taiwan University, Taipei, Taiwan; ^4^ Management Office for Health Data, China Medical University Hospital, Taichung, Taiwan; ^5^ College of Medicine, China Medical University, Taichung, Taiwan; ^6^ Department of Nursing, China Medical University Hospital, Taichung, Taiwan; ^7^ School of Medicine, Kaohsiung Medical University, Kaohsiung, Taiwan; ^8^ Department of Neurosurgery, China Medical University Hospital, Taichung, Taiwan

**Keywords:** diabetes mellitus, lumbar disk herniation, intervertebral degenerative disk disease, anti-diabetic medications, chronic back pain

## Abstract

**Background:**

Despite reports on the association between diabetes mellitus (DM) and lumbar disk herniation (LDH), large-scale, nationwide studies exploring this relationship are lacking. We aimed to examine the profiles of DM in individuals with LDH and explore the potential mechanisms underlying the development of these disorders.

**Methods:**

This retrospective, population-based study was conducted between 2008 and 2019 using data from the National Health Insurance (NHI) research database in Taiwan. The primary outcome was the date of initial LDH diagnosis, death, withdrawal from the NHI program, or end of the study period.

**Results:**

In total, 2,662,930 individuals with and 16,922,546 individuals without DM were included in this study; 719,068 matched pairs were established following propensity score matching (1:1 ratio) for sex, age, comorbidities, smoking, alcohol consumption, antihyperglycemic medications, and index year. The adjusted risk for developing LDH was 2.33-fold (95% confidence interval: 2.29−2.37; *P*<0.001), age-stratified analysis revealed a significantly greater risk of LDH in every age group, and both males and females were approximately twice as likely to develop LDH in the DM compared with non-DM cohort. Individuals with DM and comorbidities had a significantly higher risk of developing LDH than those without, and the serial models yielded consistent results. Treatment with metformin, sulfonylureas, meglitinides, thiazolidinediones, dipeptidyl peptidase-4 inhibitors, or alpha-glucosidase inhibitors was associated with a more than 4-fold increased risk of LDH in the DM cohort. DM was strongly associated with the long-term development of LDH; over the 12-year follow-up period, the cumulative risk of LDH was significantly higher in patients with than without DM (log-rank *P*<0.001).

**Conclusion:**

DM is associated with an increased risk of LDH, and advanced DM may indicate a higher risk of LDH.

## Introduction

Diabetes mellitus (DM) is a chronic disease characterized by elevated blood glucose levels, and is associated with various comorbidities. Types 1 and 2 are the two primary forms of DM, with type 2 representing approximately 90% of DM cases. Type 1 DM, also known as autoimmune DM, is a chronic disease characterized by insulin deficiency and hyperglycemia caused by the elimination of pancreatic β-cells (1). DM can affect various organ systems, resulting in severe complications over time. Individuals with type 2 DM are at risk of both microvascular and macrovascular complications, including retinopathy, nephropathy, neuropathy, and cardiovascular comorbidities. Insulin resistance and impaired insulin secretion are the primary defects of type 2 DM (2), and several antihyperglycemic medications (AHMs) with various mechanisms of action for reducing blood sugar have been developed. Commonly used oral AHMs include metformin (biguanide class), sulfonylureas (Sus), meglitinides, thiazolidinediones (TZDs), alpha-glucosidase inhibitors (Agis), dipeptidyl peptidase-4 inhibitors (DPP4is), and sodium-glucose cotransporter-2 inhibitors (SGLT2is). Injective AHMs include GLP-1 receptor agonists (GLP1Ras) and insulin.

Lumbar disk herniation (LDH) is a common cause of lower back and unilateral leg pain that commonly occurs during the fourth and fifth decades of life, affecting a significant portion of the population, with a lifetime prevalence of 10%. The occurrence of Modic changes in the lumbar region exhibited a significant increase in both the 40s and 60s (3). Similarly, the prevalence of severe intervertebral disc degeneration in the lumbar region demonstrated a significant increase in individuals aged 20s, 30s, 50s, and 70s (3). Approximately 5–20 cases of LDH per 1,000 adults occur annually, with around 95% of herniations occurring at L4-L5 or L5-S1 (4). Degeneration of intervertebral disks is a leading cause of back pain; disk degeneration, disk herniation, and radicular pain result from an imbalance between catabolic and anabolic responses (5), and disk degeneration is typically associated with herniations. Male sex, taller height, intensive work, obesity, and smoking were reported to predict LDH recurrence (6, 7), and although the relationship between DM and lumbar disk degeneration has been the subject of research, the findings remain inconsistent. Some studies have reported cases wherein DM is a risk factor in patients with multiple disk herniation. Notably, patients who underwent surgery for lumbar disk disease had a significantly higher incidence of DM than those who underwent surgery for other reasons (8). Park et al. (9) revealed that type 2 DM is significantly associated with lumbar spine disorders and frequent spinal procedures, while another study revealed a positive relationship between DM and lumbar disk diseases, including LDH (10). Additionally, a longer duration and poor control of hyperglycemia was reported to aggravate disk degeneration (11). Based on magnetic resonance imaging findings, another study found no conclusive evidence suggesting that insulin-dependent DM has a significant impact on bone density or disk degeneration (12). Therefore, whether DM is a risk factor for lumbar disk disease remains to be clarified.

Large-scale cohort studies of this topic are lacking; therefore, we aimed to delineate the association between DM and LDH by conducting a nationwide study to determine whether any difference in the risk of LDH exists between individuals with and without DM.

## Methods

### Study population

This study utilized data from the National Health Insurance Research Database (NHIRD), which is maintained by the National Health Research Institute. The NHIRD contains information from the Taiwan National Health Insurance (NHI) program, which has provided healthcare coverage to nearly all residents since its inception in 1995. By the end of 2010, the NHI program had enrolled more than 27 million people, representing approximately 99% of Taiwan’s total population. The NHIRD encrypts the identification information of each patient in the database to protect patient privacy; therefore, our investigation did not include any personal, institutional, or other data links between two or more databases. Using the International Classification of Diseases, Ninth and Tenth Revisions, Clinical Modifications (ICD-9-CM and ICD-10-CM) codes, inpatient and outpatient diagnoses were determined. This study was conducted in accordance with the principles of the Declaration of Helsinki and approved by the Institutional Review Board of China Medical University Hospital (approval number CMUH110-REC3-133(CR-1)). A waiver of informed consent was granted by the Institutional Review Board owing to the use of deidentified data in the present study. The access date to the NHIRD was on May 31, 2023.

### Study design

Patients diagnosed with either type 1 or 2 DM between 2008 and 2018 were identified in our institution’s clinical database using ICD codes (ICD-9 250; ICD-10 E08−E13). The study included Taiwanese individuals aged ≥20 years, with a study period from January 1, 2008, to December 31, 2018; patients were followed up until December 31, 2019. Accurate coding of diagnoses was ensured by requiring at least two outpatient visits or one hospitalization for inclusion. The exclusion criteria were: (1) an index date before 2008 or after 2018; (2) prior LDH diagnosis; (3) a history of kyphosis (ICD-9-CM codes 737.0, 737.1; ICD-10-CM codes M40.0−M40.3), lordosis (ICD-9-CM code 737.2; ICD-10-CM codes M40.4, M40.5), scoliosis (ICD-9-CM 737.3; ICD-10-CM M41), or spine fracture (ICD-9-CM codes 733.82, 805, 806, 808, 905, V54.8; ICD-10-CM code S32); (4) an age <20 years or >100 years; or (5) missing information regarding sex or age. The follow-up period was defined as the time between the index date (date of initial DM diagnosis) and end date (date of LDH diagnosis, or December 31, 2019, whichever occurred first). The non-DM control group was matched to individuals with DM based on enrollment criteria. This study was conducted in accordance with the Strengthening the Reporting of Observational Studies in Epidemiology (STROBE) guidelines for reporting observational studies (**Supplementary Table 1**).

### Main outcome and covariates

The main outcome of this study was the development of LDH. We censored patients on the date of the respective outcome, death, or withdrawal from the NHIRD, or at the end of follow-up on December 31, 2019, whichever came first. During the follow-up period, the incidence rates of LDH were compared between the case and control groups, with LDH defined by ICD-9-CM codes (722.10, 722.11) and ICD-10-CM codes (M51.25, M51.26, and M51.27), and DM defined by ICD-9-CM (250) and ICD-10-CM (E08–E13) codes.

To identify potential confounders that could affect LDH development, we considered sex, age, income, common comorbidities, and AHMs. The comorbidities accounted for were hypertension (ICD-9-CM codes 401−405; ICD-10-CM codes I10−I15), dyslipidemia (ICD-9-CM code 272; ICD-10-CM codes E75 and E78), chronic liver disease (CLD) (ICD-9-CM code 571; ICD-10-CM code K70, K73, and K74), chronic kidney disease (CKD) (ICD-9-CM codes 585, 586; ICD-10-CM codes N18, N19), neoplasm (ICD-9-CM code 140−239; ICD-10-CM codes C00−D49), and obesity (ICD-9-CM codes 278, 783.1; ICD-10-CM codes E66−E68 and R63.5).

Several diseases involve the bone, thereby altering the bone density of the skeletal infrastructure. Therefore, we included the following variables: bone metastasis (ICD-9-CM code 198.5; ICD-10-CM codes C79.5 and C7B.03), ankylosing spondylitis (ICD-9-CM code 720; ICD-10-CM codes M08.1, M45, M46, M48.8, and M49), and multiple myeloma (ICD-9-CM code 203; ICD-10-CM codes C88, C90, and Z51). Social behaviors, such as smoking (ICD-9-CM codes 305.1, V15.82; ICD-10-CM codes F17 and Z87.891) and alcohol consumption (ICD-9-CM code 305.0; ICD-10-CM code F10), were also included. For patients with DM, AHMs were prescribed to control blood glucose, including metformin (Anatomical Therapeutic Chemical [ATC] code A10BA02), SUs (ATC code A10BB), meglitinides (ATC code A10BX02), TZDs (ATC code A10BG), DPP4is (ATC code A10BH), SGLT2is (ATC code A10BK), AGis (ATC code A10BF01), GLP1RAs (ATC code A10BJ), and insulin (ATC code A10A).

### Statistical analysis

We used propensity score matching to reduce selection bias and improve the comparability of variables — such as sex, age, income, comorbidities, AHMs, smoking, alcohol consumption, and index year — between the DM and non-DM groups. The closest propensity score was computed, and matched pairs were created using the nearest-neighbor method, with a significance level of standardized mean difference of <0.1 indicating a significant difference between the cohorts. We used the Cox proportional hazards model to compare outcomes between the two groups, with crude and multivariate-adjusted hazard ratios (HRs) adjusted for sex, age, comorbidities, AHMs, and index year. Patients were censored if they developed LDH, died, or reached the end of the follow-up period on December 31, 2019, whichever occurred first. We performed Kaplan-Meier analysis and log-rank tests to compare the cumulative incidence of LDH between the DM and non-DM groups. Statistical analyses were performed using SAS (version 9.5; SAS Institute, Cary, NC, USA), and a two-tailed *P*-value <0.05 was considered statistically significant.

## Results

### Patient characteristics

**Figure 1:**
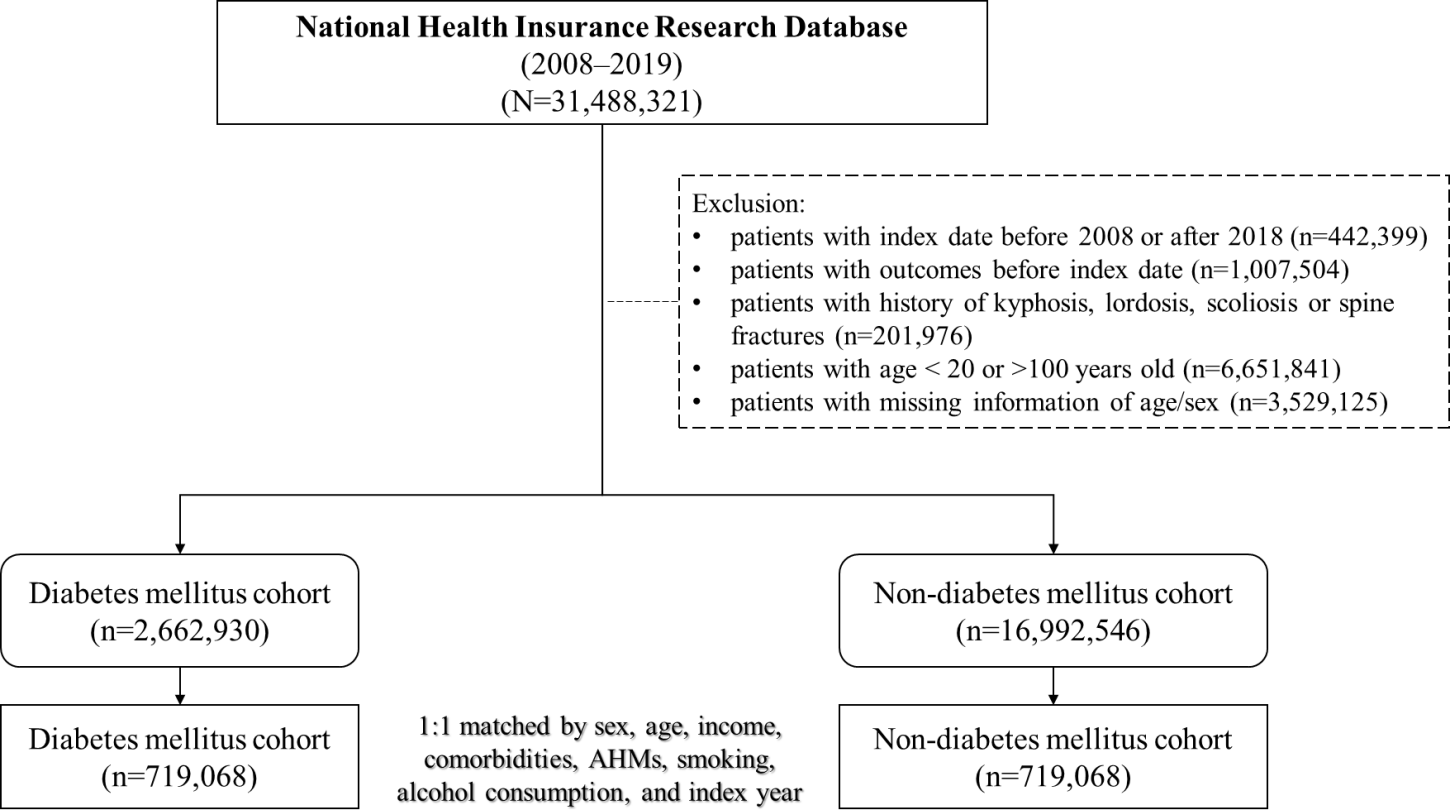
Flow chart for patients with diabetes mellitus and comparison cohort.

Among the data obtained between January 1, 2008, and December 31, 2019, we identified 31,488,321 individuals from the database. After excluding ineligible patients, we included 2,662,930 and 16,992,546 individuals in the DM and non-DM cohorts, respectively. **Figure 1** presents a flowchart of the study. We performed 1:1 propensity score matching based on the variables mentioned in the Methods section, resulting in 719,068 matched pairs of patients with and without DM. In the matched cohorts, the mean age of the DM cohort was 59.59 ± 15.32 years, and 50.27% were female; the mean follow-up duration was 5.7 ± 3.48 years. **Table 1** presents the baseline demographics of the included participants; the two cohorts showed similar baseline characteristics.

**Table 1:** Characteristics for individuals with and without diabetes mellitus.

	Non-DM		DM		
	(N=719,068)		(N=719,068)		
Variables	n	%	n	%	SMD
Sex					
Female	364088	50.63	361509	50.27	0.007
Male	354980	49.37	357559	49.73	0.007
Age					
20–39	68300	9.50	73128	10.17	0.023
40–59	285965	39.77	292912	40.74	0.02
60–79	281903	39.20	271858	37.81	0.029
≥80	82900	11.53	81170	11.29	0.008
mean, (SD)	60.09	15.17	59.59	15.32	0.033
Income					
<20001	199799	27.79	203113	28.25	0.01
20001–39999	360788	50.17	357082	49.66	0.01
>39999	158481	22.04	158873	22.09	0.001
Comorbidities					
Hypertension	367936	51.17	343790	47.81	0.067
Dyslipidemia	272958	37.96	254643	35.41	0.053
CLD	15237	2.12	11611	1.61	0.037
CKD	40519	5.63	33905	4.72	0.042
Cancer	44068	6.13	42315	5.88	0.01
Bone metastasis	26	0.00	30	0.00	0.001
AS	9044	1.26	7814	1.09	0.016
MM	138	0.02	142	0.02	<0.001
Obesity	12024	1.67	10009	1.39	0.023
Spine fractures surgery	1921	0.27	4011	0.56	0.03
Smoking	11905	1.66	10707	1.49	0.013
Alcohol consumption	15644	2.18	12682	1.76	0.03
Anti-hyperglycemic medications					
Metformin	121995	16.97	133879	18.62	0.043
SUs	31771	4.42	41406	5.76	0.061
Meglitinides	6194	0.86	8247	1.15	0.029
TZDs	4715	0.66	6662	0.93	0.031
DPP4is	22698	3.16	30507	4.24	0.058
SGLT2is	6421	0.89	8597	1.20	0.03
AGis	9338	1.30	11382	1.58	0.024
GLP1RAs	237	0.03	414	0.06	0.012
Insulin	112035	15.58	91586	12.74	0.082
Number of anti-hyperglycemic medications					
0	571979	79.54	538570	74.90	0.111
1	105712	14.70	135817	18.89	0.112
≥2	41377	5.75	44681	6.21	0.019
Follow-up years of LDH, mean (SD)	6.26	3.31	5.7	3.48	0.163

AGis, alpha-glucosidase inhibitors; AS, ankylosing spondylitis; N, number of events; CLD, chronic liver disease; CKD, chronic kidney disease; DM, diabetes mellitus; DPP4is, dipeptidyl peptidase 4 inhibitors; GLP1RAs, glucagon-like peptide 1 receptor agonists; LDH, lumbar disk herniation; MM, Multiple myeloma; SD, standard deviation; SGLT2is, sodium–glucose cotransporter 2 inhibitors; SMD, standardized mean difference; SUs, sulfonylureas; TZDs, thiazolidinediones.

### Multivariate analyses

**Figure 2:**
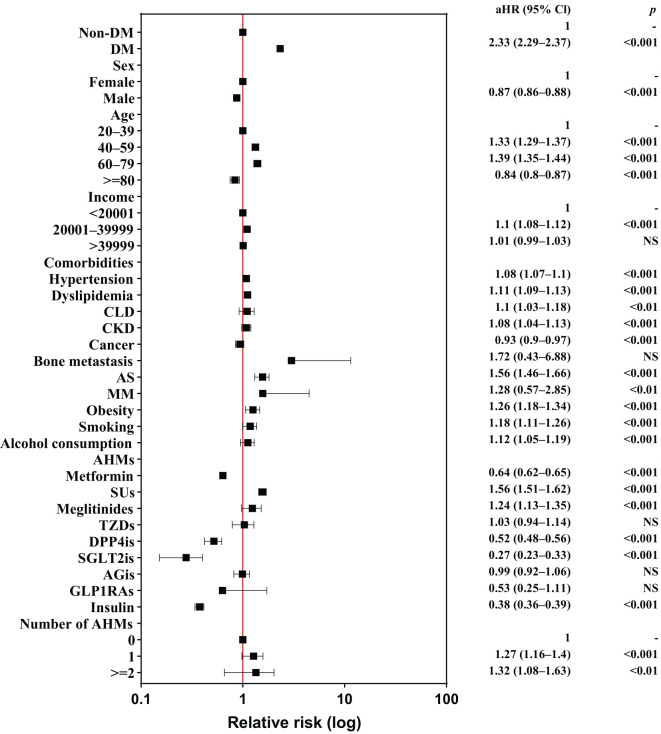
Forest plot of risk factors for lumbar disk herniation among individuals. AHMs, anti-hyperglycemic medications; aHR, adjusted hazard ratio; AGis, alpha-glucosidase inhibitors; AS, ankylosing spondylitis; CLD, chronic liver disease; CKD, chronic kidney disease; DM, diabetes mellitus; DPP4is, dipeptidyl peptidase-4 inhibitors; GLP1RAs, glucagon-like peptide-1 receptor agonists; MM, multiple myeloma; NS, nonsignificant; SGLT2is, sodium-glucose cotransporter 2 inhibitors; SUs, sulfonylureas; TZDs, thiazolidinediones; 95% CI, 95% confidence interval.

**Figure 2** shows a forest plot of the risk factors for LDH in individuals with DM; **Supplementary Table 2** summarizes these risk factors. In the multivariable Cox regression analysis, 20,729 (2.88%) patients with LDH did not have a previous diagnosis of DM, and 45,243 (6.29%) patients with LDH were diagnosed with DM before the occurrence of LDH (incidence rate: 4.6 *vs*. 11.0 per 1,000 person-years). The crude HR (cHR) of DM was 2.38 (95% confidence interval [CI], 2.34−2.42, *P*<0.001) in patients with LDH. Individuals with DM had a higher risk of developing LDH than those without DM after adjusting for sex, age, comorbidities and AHMs (adjusted HR [aHR], 2.33; 95% CI, 2.29–2.37; *P*<0.001). The risk of LDH was found to increase in individuals aged 40–59 years and 60–79 years when compared with individuals aged 20–39 years, with aHRs of 1.33 and 1.39, respectively. However, this risk decreased in individuals aged >80 years. Males had a significantly lower risk of LDH (aHR, 0.87; 95% CI, 0.86–0.88; *P*<0.001) than females, and income level was not associated with LDH. Patients with comorbidities — such as hypertension, dyslipidemia, CLD, CKD, obesity, smoking, and alcohol consumption — had a significantly higher risk of LDH, whereas those with cancer had a significantly lower risk of LDH. Notably, patients with AS had a significantly elevated risk of LDH (aHR, 1.56; 95% CI, 1.46–1.66; *P*<0.001). Participants using 1 or >2 AHMs had a prominent risk of LDH (aHR, 1.27 and 1.32, respectively).

**Figure 3:**
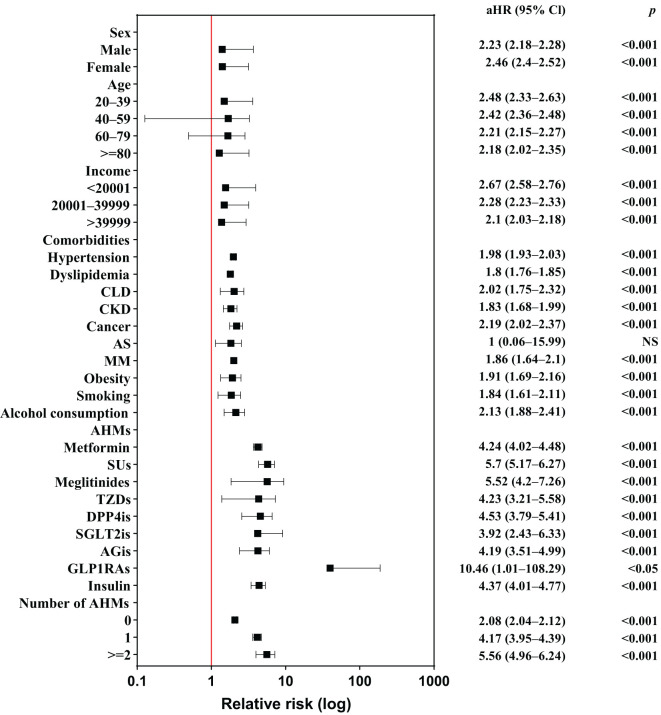
Forest plot of risk factors for lumbar disk herniation among individuals with and without diabetes mellitus. AHMs, anti-hyperglycemic medications; aHR, adjusted hazard ratio; AGis, alpha-glucosidase inhibitors; AS, ankylosing spondylitis; CLD, chronic liver disease; CKD, chronic kidney disease; DPP4is, dipeptidyl peptidase-4 inhibitors; GLP1RAs, glucagon-like peptide-1 receptor agonists; MM, multiple myeloma; NS, nonsignificant; SGLT2is, sodium–glucose cotransporter 2 inhibitors; SUs, sulfonylureas; TZDs, thiazolidinediones; 95% Cl, 95% confidence interval.

**Figure 3** presents a forest plot of the risk factors for LDH in individuals with and without DM; **Supplementary Table 3** summarizes these findings. Individuals with DM had a significantly higher risk of developing LDH, regardless of sex, age, income level, or the coexistence of any comorbidity. Patients using AHMs have a more prominent risk of LDH than those who do not; notably, individuals receiving GLP1RAs had 10.46-fold higher risk of developing LDH than those did not (95% CI, 1.01−108.29; *P*=0.0489). The use of metformin, SUs, meglitinides, TZDs, DPP4is, or AGis was associated with a more than 4-fold increased risk of LDH in patients with than without DM, and the number of AHMs used was positively associated with the risk of LDH development.

### Stratified analyses

To investigate the effect of covariates, four models were used to determine the risk of LDH in patients both with and without DM. **Table 2** presents the HRs and 95% CIs for the two cohorts, as well as each model. In Model 1, the cHR was examined. In models 2–4, the aHRs were obtained based on adjustments to different variables.

**Table 2:** Hazard ratios and 95% confidence intervals for lumbar disk herniation in different models.

	Non-DM	DM
LDH		
Number of events	20,729	45,243
PY	4,499,516	4,102,043
IR	4.61	11.03
Model1. cHR (95% CI)	(Reference)	2.38 (2.34, 2.42)***
Model2. aHR (95% CI)	(Reference)	2.37 (2.33, 2.41)***
Model3. aHR (95% CI)	(Reference)	2.38 (2.34, 2.42)***
Model4. aHR (95% CI)	(Reference)	2.33 (2.29, 2.37)***

Model2: adjusted to sex and age.

Model3: adjusted to Model2 (sex, age), and comorbidities.

Model4: adjusted to Model3 (sex, age, comorbidities), and anti-hyperglycemic medications.

aHR, adjusted hazard ratio; cHR, crude hazard ratio; DM, diabetes mellitus; IR, incidence rate per 1,000 person-years; LDH, lumbar herniation; PY, person-years. ***P <0.001.

### Duration analysis

**Table 3** presents the risk of LDH in both cohorts according to the duration of LDH diagnosis. Regarding follow-up periods <4 years, the aHR of LDH development in the DM cohort was 2.6 (95% Cl, 2.54−2.66; *P*<0.001). After >10 years of follow-up, the risk of LDH in the DM cohort remained significantly higher than in the non-DM cohort (aHR, 1.51; 95% CI, 1.37−1.68; *P*<0.001).

**Table 3:** The risks of lumbar disk herniation in the diabetes mellitus cohort relative to the non-diabetes mellitus cohort in terms of different follow-up period.

	Non-DM			DM						
Follow-up years	n	PY	IR	n	PY	IR	cHR	(95% CI)	aHR	(95% CI)
<4	11501	2008268	5.73	29605	1897823	15.60	2.69	(2.63, 2.74)***	2.6	(2.54, 2.66)***
4–7	5664	1401677	4.04	10223	1253535	8.16	2.02	(1.95, 2.08)***	1.96	(1.89, 2.02)***
8–10	2919	822924	3.55	4524	714300	6.33	1.8	(1.72, 1.88)***	1.76	(1.68, 1.84)***
>10	645	266647	2.42	891	236384	3.77	1.52	(1.37, 1.68)***	1.51	(1.37, 1.68)***

aHR, adjusted hazard ratio; cHR, crude hazard ratio; DM, diabetes mellitus; IR, incidence rate per 1,000 person-years; PY, person-years.

†: adjusted by sex, age, comorbidities, and anti-hyperglycemic medications. ***P <0.001.

### Cumulative incidence of LDH

**Figure 4:**
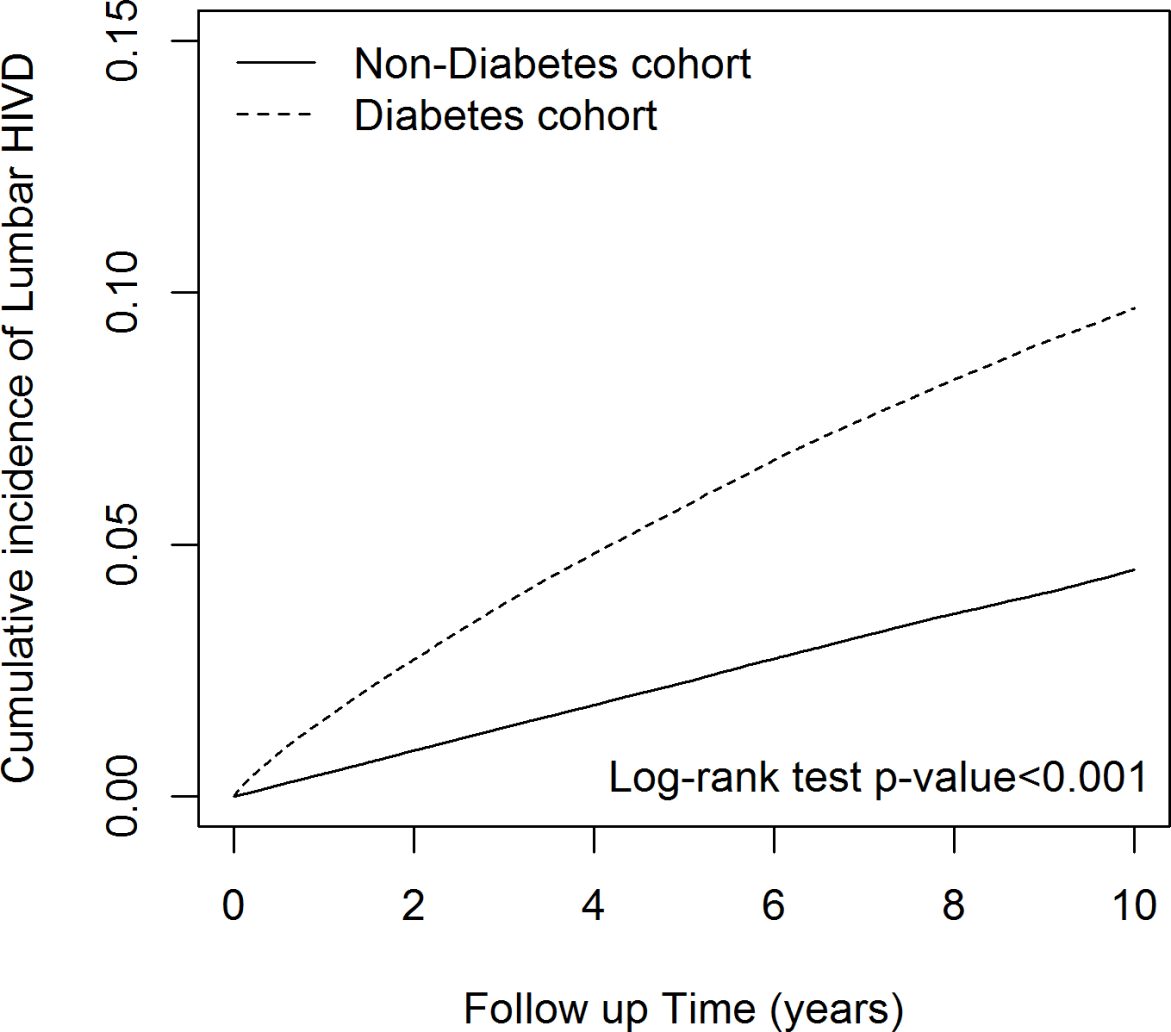
The cumulative incidence of lumbar disk herniation in diabetes mellitus cohort and control cohort.

**Figure 4** illustrates the Kaplan-Meier cumulative incidence of LDH, which was significantly higher in the DM than non-DM cohort (log-rank *P*<0.001).

## Discussion

In recent years, there has been a notable increase in the occurrence of both type 1 and type 2 DM, suggesting that a large proportion of the population faces challenges and complications associated with this chronic condition. A study conducted in this context revealed that individuals with DM displayed elevated levels of LDH. These results suggest that inadequate long-term management of DM may contribute to the development of LDH, and potentially increase the chances of requiring surgical intervention.

Degenerative disk disease poses a significant healthcare issue, leading to persistent and often intense back pain that has a detrimental impact on the patient’s wellbeing, and contributes to rising healthcare expenses. Understanding the risk factors associated with lumbar disk degeneration is crucial to implementing strategies that prevent or slow disease development and progression. Recent studies indicate a higher vulnerability to intervertebral disk disease in females compared with males; still, the specific impact of DM on intervertebral disk degeneration based on differences in sex remains unclear (13). Our study reported consistent findings that males bear a lower risk of LDH than females (aHR, 0.87; 95% Cl, 0.86−0.88). Several clinical studies have demonstrated that the incidence of intervertebral disk disease is higher in individuals with obesity and DM (14); notably, growing evidence indicates a correlation between a high body mass index (BMI), obesity, or overweight, and an increased risk of intervertebral disk degeneration (15). Özcan-Ekş et al. (16) found that severe intervertebral disc disease was significantly more prevalent in obese individuals compared to non-obese individuals, with a prevalence rate of 73.5% in obese patients compared to 50.4% in non-obese patients. In addition, there was a higher likelihood of obese patients exhibiting Modic changes at any lumbar level, particularly in women. This result corroborates our research outcomes. In the present study, obesity was found to increase the risk of LDH (aHR, 1.12; 95% Cl, 1.05−1.19).

Associations were also found between dyslipidemia and LDH levels (aHR, 1.11; 95% Cl, 1.09−1.13); however, the relationship between serum lipid levels and back pain remains under debate. Some theories propose that advanced atherosclerosis may play a role in microvessel disease and spinal disk degeneration. Abnormal lipid levels have also been suggested as a potential mechanism that leads to atherosclerosis in the blood vessels of the lumbar region, which in turn can cause low back pain. Additionally, individuals with high TG levels were more likely to experience disk herniation (odds ratio, 2.974; 95% CI, 1.488–5.945) (17). The age-adjusted prevalence of low back pain was inversely associated with HDL cholesterol levels, and positively associated with triglyceride; however, after accounting for age, the total cholesterol levels were not significantly associated with low back pain in either gender (18). Cholesterol levels are also associated with CBP in patients with DM; elevated LDL cholesterol levels were associated with CBP, whereas elevated HDL cholesterol levels were negatively associated.

Recently, smoking was shown to negatively influence LDH levels, likely due to microangiopathy. In the present study, we found that smokers had a greater probability of suffering from LDH (aHR, 1.18; 95% Cl, 1.11−1.26) compared with nonsmokers. Two potential mechanisms for disk degeneration caused by smoking have been postulated: (1) downregulation of glycosaminoglycan biosynthesis and cell proliferation mediated by nicotine, and (2) decreased supply of nutrients to the intervertebral disk. The results of our study align with those of previous studies that established a correlation between DM and degenerative disk diseases (9, 19). Furthermore, previous investigations demonstrated that individuals with DM tend to experience worse outcomes after lumbar discectomy than nondiabetic controls, including higher rates of reoperation and longer hospital stays (20).

Elevated preoperative HbA1c levels and long-term DM are risk factors for unfavorable outcomes following cervical laminoplasty in patients with DM and cervical spondylotic myelopathy (21). In a review of patients who underwent discectomy for LDH, Mobbs et al. (20) reported higher rates of LDH recurrence and reoperation in patients with DM (28%) than in controls (3.5%). However, Vogt et al. (22) did not find a correlation between a history of DM and the prevalence of L4–L5 degenerative spondylolisthesis. A duration >10 years and poor control of type 2 DM are risk factors for lumbar disk degeneration, with a longer duration associated with more severe disk degeneration (23). The duration of DM is also associated with the need for spinal surgery, suggesting that the cumulative effects of DM over time may contribute to degenerative changes requiring surgical intervention. Lumbar degenerative disk disease is associated with male sex, HbA1c levels, and venous glucose (24). This study also reported a potential link between DM and lumbar spinal stenosis. A Mendelian randomization analysis revealed a causal effect of type 2 DM on degenerative disk disease that persisted even when adjusted to BMI (25). Magnetic resonance imaging revealed a strong correlation between the severity and duration of DM and the presence of Modic changes (26). DM is also associated with poor outcomes following lumbar discectomy and cervical laminoplasty; a meta-analysis demonstrated that DM increased the risk of postoperative mortality, surgical site infection, deep venous thrombosis, and prolonged hospitalization after spinal surgery (27).

A positive correlation has been identified between DM and degenerative lumbar disk disease; high preoperative HbA1c levels and long-term DM are risk factors for poor cervical laminoplasty outcomes in patients with DM and cervical spondylotic myelopathy (21). Several studies have also suggested that hyperglycemia promotes the formation of advanced glycation end products in the nucleus pulposus, which contributes to the progression of disk degeneration. Recent animal studies have examined the association between hyperglycemia and intervertebral disk degeneration. An animal study indicated that DM accelerates disk degeneration through microangiopathy (28); additionally, microvascular disease — a characteristic of DM — may impair disk nutrition and contribute to degeneration. In a study using a rat model, hyperglycemia stimulated disk autophagy — a process of cellular self-degradation — and accelerated stress-induced senescence in nucleus pulposus cells. Autophagy in nucleus pulposus and annulus fibrosus cells also appears to play a significant role in lumbar degenerative diseases. Two studies have demonstrated that high glucose-induced oxidative stress accelerates premature stress-induced senescence in young rat annulus fibrosis cells (29, 30).

The evaluation of proteoglycans in the intervertebral disks of individuals with DM has revealed a reduction in sulfate incorporation into glycosaminoglycan molecules, and lower rates of glycosylation. These findings align with those of a previous study conducted by Robinson et al. (31), who observed a lower presence of proteoglycans in the intervertebral disks of patients with than without DM. These variations may contribute to elevated vulnerability to recurrent herniation in individuals with DM, as sulfation and proteoglycans are recognized for their role in reinforcing the collagen matrix of the disk. Nevertheless, despite the histological evidence, clinical studies have not established a conclusive association between DM and the rate of recurrent LDH.

Notably, the administration of AHMs, such as SUs or meglitinides, was found to be significantly associated with an increased risk of LDH; additionally, the simultaneous use of multiple AHMs, which suggests inadequate blood sugar control, was significantly associated with an increased risk of LDH. Conversely, the use of metformin, DPP4is, SGLT2is, or insulin was significantly associated with a lower risk of LDH. No significant association with LDH was observed for TZDs, AGis, or GLP1RAs. In multivariate analyses, patients with DM using any AHM exhibited a higher risk of LDH than those without DM. This suggests that worsening hyperglycemia, which requires medication, is associated with an increased risk of LDH.

This study demonstrated that participants who used AHMs were at a higher risk of LDH than those who did not. Furthermore, patients who were coadministered >2 AHMs were at a significantly higher risk of developing LDH than those who were coadministered <2 AHMs. These findings suggest that patients with poorly controlled DM tend to exhibit more severe disk degeneration than those with adequate control, as well as that DM is a risk factor for LDH, with an effect dependent on the duration and level of disease control. Additionally, the study observed that the use of medications like metformin, DPP4is, SGLT2is, or exogenous insulin was associated with a lower incidence of LDH.

DM is a complex condition that likely contributes to LDH via various mechanisms; furthermore, the correlation between AHMs and LDH can vary depending on specific clinical circumstances. Therefore, maintaining strict blood glucose control is crucial for preventing or delaying lumbar degenerative diseases in older patients with DM (30). This study acknowledges the importance of further investigation to understand the mechanisms underlying the association between DM and LDH, as well as the disease burden of DM in spinal pathologies; thus, further prospective comparative studies with longer follow-up periods are required to confirm our results.

This study has some limitations, including its retrospective cohort design; however, observational studies cannot provide insight into the causal relationship between DM and interverbal degenerative disk disease, even when based on larger sample sizes. Second, the NHIRD lacks relevant clinical and laboratory information, such as BMI, lipid profiles, and HbA1c levels. Third, although we adjusted for various confounding factors, the residual confounding factors may have biased our results; cohort studies are usually associated with bias due to uncovered and unobserved confounding factors. Last, our findings only be related to the Taiwanese population; thus, similar studies should be performed in different countries to determine whether our observations apply to other populations. Despite the notable limitations mentioned above, the primary objective of this study was to evaluate the overall correlation between DM burden and LDH levels. However, to delve into more precise inquiries, future investigations should consider conducting smaller and more targeted studies.

## Conclusions

In recent years, the prevalence of both type 1 and type 2 DM has increased in children and adolescents, indicating that a growing population is at risk of complications associated with this chronic disease. This study aimed to explore the relationship between DM and LDH. These findings revealed that higher LDH burden metrics were identified in patients with than without DM, suggesting that advanced DM contributes to the development of LDH. Elevated blood sugar levels, modified proteoglycan composition, microvascular diseases, and cholesterol levels are potential factors involved in the mechanisms underlying LDH in individuals with DM. In conclusion, early and strict blood glucose control is important to prevent the development of lumbar degenerative diseases in patients with DM.

## IRB approval status

This study was reviewed and approved by the Institutional Review Board of China Medical University Hospital (ID number CMUH110-REC3-133(CR-1)).

## Impact statement

Diabetes mellitus contributes to the development of lumbar disk herniation; thus, early and strict blood glucose control is important to prevent the development of lumbar degenerative diseases in these patients.

## Data availability statement

The original contributions presented in the study are included in the article/**Supplementary Material**. Further inquiries can be directed to the corresponding author.

## Ethics statement

The studies involving humans were approved by Institutional Review Board of China Medical University Hospital. The studies were conducted in accordance with the local legislation and intitutional requirements. Written informed consent for participation was not required from the participants or the participants' legal guardians because only deidentified data was obtained and used in the present study.

## Author contributions

J-XL: Conceptualization, Investigation, Methodology, Visualization, Writing – original draft, Writing – review & editing, Software. T-JH: Data curation, Formal Analysis, Methodology, Software, Writing – review & editing. S-BH: Data curation, Project administration, Software, Validation, Writing – review & editing. Y-HL: Project administration, Supervision, Validation, Writing – review & editing.
