# Supplementary material for: Strong association of lumbar disk herniation with diabetes mellitus: a 12-year nationwide retrospective cohort study
Source: Front Endocrinol (Lausanne). 2023 Nov 2;14:1260566. doi: 10.3389/fendo.2023.1260566 (PMC10652879; doi:10.3389/fendo.2023.1260566)
Supplement: Supplementary file 1 [file DataSheet_1.pdf]

Supplementary Material

Study title:
Strong association of lumbar disk herniation with diabetes mellitus: A 12-year nationwide retrospective cohort study.

Supplementary Table 1. Strengthening the reporting of observational study checklist for the present study.
Supplementary Table 2. Incidence and hazards of lumbar disk herniation among study individuals.
Supplementary Table 3. Incidence and hazards of lumbar disk herniation in individuals with and without diabetes mellitus.

Supplementary Table 1. Strengthening the reporting of observational study checklist for the present study.

	Item No.	Recommendation	Page No.	Relevant text from manuscript
**Title and abstract**	1	(*a*) Indicate the study's design with a commonly used term in the title or the abstract	Title	
		(*b*) Provide in the abstract an informative and balanced summary of what was done and what was found	Abstract	
**Introduction**				
Background/rationale	2	Explain the scientific background and rationale for the investigation being reported	Introduction, paragraph 1 to 3	
Objectives	3	State specific objectives, including any prespecified hypotheses	Introduction, paragraph 4	
**Methods**				
Study design	4	Present key elements of study design early in the paper	Methods, Study design	
Setting	5	Describe the setting, locations, and relevant dates, including periods of recruitment, exposure, follow-up, and data collection	Methods, Study population	
Participants	6	(*a*) *Cohort study*—Give the eligibility criteria, and the sources and methods of selection of participants. Describe methods of follow-up<br>*Case-control study*—Give the eligibility criteria, and the sources and methods of case ascertainment and control selection. Give the rationale for the choice of cases and controls<br>*Cross-sectional study*—Give the eligibility criteria, and the sources and methods of selection of participants	Methods, Study population	
		(*b*) *Cohort study*—For matched studies, give matching criteria and number of exposed and unexposed<br>*Case-control study*—For matched studies, give matching criteria and the number of controls per case	NA	
Variables	7	Clearly define all outcomes, exposures, predictors, potential confounders, and effect modifiers. Give diagnostic criteria, if applicable	Methods, Main outcome and covariates	
Data sources/ measurement	8*	For each variable of interest, give sources of data and details of methods of assessment (measurement). Describe comparability of assessment methods if there is more than one group	Methods, Main outcome and covariates	
Bias	9	Describe any efforts to address potential sources of bias	Methods, Main outcome and covariates	
Study size	10	Explain how the study size was arrived at	Figure 1	
Quantitative variables	11	Explain how quantitative variables were handled in the analyses. If applicable, describe which groupings were chosen and why	Methods, Main outcome and covariates	
Statistical methods	12	(*a*) Describe all statistical methods, including those used to control for confounding	Methods, Statistical analysis	

		(*b*) Describe any methods used to examine subgroups and interactions	Methods, Main outcome and covariates
		(*c*) Explain how missing data were addressed	Methods, Study design
		(*d*) *Cohort study*—If applicable, explain how loss to follow-up was addressed<br>*Case-control study*—If applicable, explain how matching of cases and controls was addressed<br>*Cross-sectional study*—If applicable, describe analytical methods taking account of sampling strategy	Methods, Main outcome and covariates
		(*e*) Describe any sensitivity analyses	NA
**Results**			
Participants	13*	(a) Report numbers of individuals at each stage of study—eg numbers potentially eligible, examined for eligibility, confirmed eligible, included in the study, completing follow-up, and analyzed	Results, Patient characteristics
		(b) Give reasons for non-participation at each stage	Results, Patient characteristics
		(c) Consider use of a flow diagram	NA
Descriptive data	14*	(a) Give characteristics of study participants (eg demographic, clinical, social) and information on exposures and potential confounders	Results, Patient characteristics
		(b) Indicate number of participants with missing data for each variable of interest	NA
		(c) *Cohort study*—Summarise follow-up time (eg, average and total amount)	NA
Outcome data	15*	*Cohort study*—Report numbers of outcome events or summary measures over time	Results, Multivariate analyses
		Case-control study—Report numbers in each exposure category, or summary measures of exposure	
		Cross-sectional study—Report numbers of outcome events or summary measures	
Main results	16	(*a*) Give unadjusted estimates and, if applicable, confounder-adjusted estimates and their precision (eg, 95% confidence interval). Make clear which confounders were adjusted for and why they were included	Results, Multivariate analyses
		(*b*) Report category boundaries when continuous variables were categorized	NA
		(*c*) If relevant, consider translating estimates of relative risk into absolute risk for a meaningful time period	NA
Other analyses	17	Report other analyses done—eg analyses of subgroups and interactions, and sensitivity analyses	Results, Multivariate analyses
**Discussion**			
Key results	18	Summarise key results with reference to study objectives	Discussion, paragraph 1 to 9
Limitations	19	Discuss limitations of the study, taking into account sources of potential bias or imprecision. Discuss both direction and magnitude of any potential bias	Discussion, paragraph 10

Interpretation	20	Give a cautious overall interpretation of results considering objectives, limitations, multiplicity of analyses, results from similar studies, and other relevant evidence	Discussion, paragraph 1 to 9
Generalizability	21	Discuss the generalizability (external validity) of the study results	NA
**Other information**			
Funding	22	Give the source of funding and the role of the funders for the present study and, if applicable, for the original study on which the present article is based	NA

*Give information separately for cases and controls in case-control studies and, if applicable, for exposed and unexposed groups in cohort and cross-sectional studies. NA, not applicable.

Supplementary Table 2. Incidence and hazards of lumbar disk herniation among study individuals.

	LDH			Cox proportional hazards model			
	Event	PY	IR	cHR	95% CI	aHR†	95% CI
Non-DM	20729	4499516	4.6069	1.00	(Reference)	1.00	(Reference)
DM	45243	4102043	11.0294	2.38	(2.34, 2.42)***	2.33	(2.29, 2.37)***
Sex							
Female	36989	4454573	8.3036	1.00	(Reference)	1.00	(Reference)
Male	28983	4146987	6.9889	0.84	(0.82, 0.85)***	0.87	(0.86, 0.88)***
Age							
20–39	5158	955135	5.4003	1.00	(Reference)	1.00	(Reference)
40–59	30311	3762678	8.0557	1.48	(1.44, 1.53)***	1.33	(1.29, 1.37)***
60–79	27366	3201864	8.5469	1.56	(1.51, 1.6)***	1.39	(1.35, 1.44)***
≥80	3137	681882	4.6005	0.81	(0.77, 0.85)***	0.84	(0.8, 0.87)***
Income							
<20001	16323	2318526	7.0403	1.00	(Reference)	1.00	(Reference)
20001–39999	34879	4308414	8.0956	1.15	(1.13, 1.17)***	1.1	(1.08, 1.12)***
>39999	14770	1974620	7.4799	1.07	(1.04, 1.09)***	1.01	(0.99, 1.03)
Comorbidities							
Hypertension	31450	3808476	8.2579	1.12	(1.1, 1.13)***	1.08	(1.07, 1.1)***
Dyslipidemia	25941	2865297	9.0535	1.27	(1.25, 1.29)***	1.11	(1.09, 1.13)***
CLD	840	106650	7.8762	0.98	(0.92, 1.05)	1.1	(1.03, 1.18)**
CKD	2275	309851	7.3422	0.91	(0.88, 0.95)***	1.08	(1.04, 1.13)***
Cancer	2766	391412	7.0667	0.89	(0.86, 0.93)***	0.93	(0.9, 0.97)***
Bone metastasis	<3	<3	6.26	1	(0.26, 3.88)	1.72	(0.43, 6.88)
AS	1043	81081	12.8636	1.63	(1.54, 1.74)***	1.56	(1.46, 1.66)***
MM	6	756	7.9322	0.96	(0.43, 2.14)	1.28	(0.57, 2.85)
Obesity	1068	110777	9.641	1.22	(1.15, 1.3)***	1.26	(1.18, 1.34)***
Smoking	902	94764	9.5184	1.18	(1.11, 1.26)***	1.18	(1.11, 1.26)***
Alcohol consumption	1058	128341	8.2437	1.04	(0.98, 1.1)	1.12	(1.05, 1.19)***
AHMs							
Metformin	8036	1630137	4.9296	0.6	(0.58, 0.61)***	0.64	(0.62, 0.65)***
SUs	3972	461626	8.6044	1.14	(1.1, 1.17)***	1.56	(1.51, 1.62)***
Meglitinides	483	76051	6.351	0.82	(0.75, 0.9)***	1.24	(1.13, 1.35)***
TZDs	423	74615	5.6691	0.74	(0.68, 0.82)***	1.03	(0.94, 1.14)
DPP4is	950	325866	2.9153	0.37	(0.35, 0.4)***	0.52	(0.48, 0.56)***
SGLT2is	114	90662	1.2574	0.16	(0.14, 0.2)***	0.27	(0.23, 0.33)***
AGis	892	135396	6.5881	0.86	(0.81, 0.92)***	0.99	(0.92, 1.06)
GLP1RAs	7	3996	1.7515	0.23	(0.11, 0.49)***	0.52	(0.25, 1.08)
Insulin	2734	1006765	2.7156	0.32	(0.31, 0.33)***	0.37	(0.36, 0.39)***
Number of AHMs							
0	53746	6554450	8.20	1.00	-	-	(Reference)
1	10017	1498787	6.68	0.82	(0.8, 0.84)***	1.27	(1.16, 1.4)***
≥2	2209	548322	4.03	0.49	(0.47, 0.52)***	1.32	(1.08, 1.63)**

AHMs, anti-hyperglycemic medications; aHR, adjusted hazard ratio; AGis, alpha-glucosidase inhibitors; AS, ankylosing spondylitis; cHR, crude hazard ratio; CLD, chronic liver disease; CKD, chronic kidney disease; DM; diabetes mellitus; DPP4is, dipeptidyl peptidase-4 inhibitors; GLP1RAs, glucagon-like peptide-1 receptor agonists; IR, incidence rate, per 1,000 person-year; LDH, lumbar disc herniation; MM, multiple myeloma; N, number of events; PY, person-year; SGLT2is, sodium–glucose cotransporter 2 inhibitors; SUs, sulfonylureas; TZDs, thiazolidinediones; 95% Cl, 95% confidence interval.

† Adjusted hazard ratio estimated by multivariable analysis including sex, age, comorbidities, and anti-hyperglycemic medications.

***P* <0.01, ****P* <0.001.

In accordance with the guidelines of the National Health Insurance Research Database for safeguarding patient privacy, the data retrieval from the database is not possible as the event count is less than three. Therefore, it will be indicated as "<3" to ensure confidentiality.

Supplementary Table 3. Incidence and hazards of lumbar disk herniation in individuals with and without diabetes mellitus.

	Non-DM			DM			LDH					
	Event	PY	IR	Event	PY	IR	cHR	95% CI	*P*	aHR[†]	95% CI	*P*
Sex												
Female	11971	2325797	5.15	25018	2128776	11.75	2.27	(2.22, 2.32)***	<0.001	2.23	(2.18, 2.28)***	<0.001
Male	8758	2173720	4.03	20225	1973267	10.25	2.53	(2.46, 2.59)***	<0.001	2.46	(2.4, 2.52)***	<0.001
Age												
20–39	1516	482717	3.14	3642	472418	7.71	2.44	(2.3, 2.59)***	<0.001	2.48	(2.33, 2.63)***	<0.001
40–59	9107	1924608	4.73	21204	1838071	11.54	2.43	(2.37, 2.49)***	<0.001	2.42	(2.36, 2.48)***	<0.001
60–79	9008	1700052	5.3	18358	1501812	12.22	2.3	(2.24, 2.35)***	<0.001	2.21	(2.15, 2.27)***	<0.001
≥ 80	1098	392140	2.8	2039	289742	7.04	2.43	(2.25, 2.61)***	<0.001	2.18	(2.02, 2.35)***	<0.001
Income												
<20001	4698	1230069	3.82	11625	1088456	10.68	2.77	(2.68, 2.86)***	<0.001	2.67	(2.58, 2.76)***	<0.001
20001–39999	11140	2254635	4.94	23739	2053779	11.56	2.33	(2.28, 2.38)***	<0.001	2.28	(2.23, 2.33)***	<0.001
>39999	4891	1014812	4.82	9879	959808	10.29	2.13	(2.06, 2.2)***	<0.001	2.1	(2.03, 2.18)***	<0.001
Comorbidities												
Hypertension												
No	9525	2460869	3.87	24997	2332214	10.72	2.75	(2.68, 2.81)***	<0.001	2.71	(2.64, 2.77)***	<0.001
Yes	11204	2038647	5.5	20246	1769829	11.44	2.07	(2.03, 2.12)***	<0.001	1.98	(1.93, 2.03)***	<0.001
Dyslipidemia	.	.	.	.	.	.						
No	11027	2993825	3.68	29004	2742437	10.58	2.85	(2.79, 2.91)***	<0.001	2.78	(2.72, 2.84)***	<0.001
Yes	9702	1505691	6.44	16239	1359606	11.94	1.85	(1.8, 1.9)***	<0.001	1.8	(1.76, 1.85)***	<0.001
CLD	.	.	.	.	.	.						
No	20388	4435606	4.6	44744	4059303	11.02	2.38	(2.34, 2.42)***	<0.001	2.33	(2.29, 2.37)***	<0.001
Yes	341	63910	5.34	499	42740	11.68	2.19	(1.9, 2.51)***	<0.001	2.02	(1.75, 2.32)***	<0.001
CKD	.	.	.	.	.	.						
No	19775	4317670	4.58	43922	3974038	11.05	2.4	(2.36, 2.44)***	<0.001	2.35	(2.31, 2.39)***	<0.001
Yes	954	181846	5.25	1321	128005	10.32	1.96	(1.8, 2.13)***	<0.001	1.83	(1.68, 1.99)***	<0.001
Cancer	.	.	.	.	.	.						
No	19776	4282495	4.62	43430	3927652	11.06	2.38	(2.34, 2.42)***	<0.001	2.33	(2.29, 2.37)***	<0.001
Yes	953	217021	4.39	1813	174391	10.40	2.35	(2.17, 2.54)***	<0.001	2.19	(2.02, 2.37)***	<0.001
Bone metastasis												
No	NA	NA	4.61	NA	NA	11.03	2.38	(2.34, 2.42)***	<0.001	2.33	(2.29, 2.37)***	<0.001
Yes	NA	NA	8.23	NA	NA	16.86	2.14	(0.13, 34.23)	0.5913	1	(0.06, 15.99)	1
AS	.	.	.	.	.	.						
No	20314	4454228	4.56	44615	4066250	10.97	2.39	(2.35, 2.43)***	<0.001	2.34	(2.3, 2.38)***	<0.001
Yes	415	45288	9.16	628	35793	17.55	1.89	(1.67, 2.14)***	<0.001	1.86	(1.64, 2.1)***	<0.001
MM	.	.	.	.	.	.						
No	20729	4499029	4.61	45237	4101774	11.03	2.38	(2.34, 2.42)***	<0.001	2.33	(2.29, 2.37)***	<0.001
Yes	0	488	0	6	269	22.32	NA	NA	NA	NA	NA	NA
Obesity	.	.	.	.	.	.						
No	20279	4437190	4.57	44625	4053592	11.01	2.4	(2.36, 2.44)***	<0.001	2.34	(2.3, 2.37)***	<0.001
Yes	450	62326	7.22	618	48451	12.76	1.75	(1.55, 1.98)***	<0.001	1.91	(1.69, 2.16)***	<0.001
Smoking	.	.	.	.	.	.						
No	20372	4448140	4.58	44698	4058655	11.01	2.39	(2.35, 2.43)***	<0.001	2.34	(2.3, 2.37)***	<0.001
Yes	357	51376	6.95	545	43388	12.56	1.8	(1.58, 2.06)***	<0.001	1.84	(1.61, 2.11)***	<0.001
Alcohol consumption												
No	20322	4425810	4.59	44592	4047409	11.02	2.39	(2.35, 2.43)***	<0.001	2.33	(2.29, 2.37)***	<0.001
Yes	407	73706	5.52	651	54635	11.92	2.15	(1.9, 2.44)***	<0.001	2.13	(1.88, 2.41)***	<0.001
AHMs												
Metformin												
No	19153	3676987	5.21	38783	3294435	11.77	2.25	(2.21, 2.29)***	<0.001	2.17	(2.13, 2.2)***	<0.001
Yes	1576	822529	1.92	6460	807608	8.00	4.18	(3.96, 4.42)***	<0.001	4.24	(4.02, 4.48)***	<0.001
SUs	.	.	.	.	.	.						
No	20256	4284742	4.73	41744	3855191	10.83	2.28	(2.24, 2.32)***	<0.001	2.24	(2.2, 2.28)***	<0.001
Yes	473	214774	2.2	3499	246852	14.17	6.33	(5.75, 6.97)***	<0.001	5.7	(5.17, 6.27)***	<0.001
Meglitinides	.	.	.	.	.	.						
No	20669	4463602	4.63	44820	4061906	11.03	2.37	(2.33, 2.41)***	<0.001	2.32	(2.28, 2.36)***	<0.001
Yes	60	35914	1.67	423	40137	10.54	6.24	(4.76, 8.17)***	<0.001	5.52	(4.2, 7.26)***	<0.001
TZDs	.	.	.	.	.	.						
No	20669	4467047	4.63	44880	4059898	11.05	2.38	(2.34, 2.42)***	<0.001	2.32	(2.28, 2.36)***	<0.001
Yes	60	32469	1.85	363	42145	8.61	4.65	(3.54, 6.12)***	<0.001	4.23	(3.21, 5.58)***	<0.001

DPP4is	.	.	.	.	.	.						
No	20585	4353107	4.73	44437	3922587	11.33	2.38	(2.34, 2.42)***	<0.001	2.31	(2.27, 2.35)***	<0.001
Yes	144	146409	0.98	806	179456	4.49	4.61	(3.86, 5.51)***	<0.001	4.53	(3.79, 5.41)***	<0.001
SGLT2is	.	.	.	.	.	.						
No	20708	4457647	4.65	45150	4053251	11.14	2.38	(2.35, 2.42)***	<0.001	2.33	(2.29, 2.36)***	<0.001
Yes	21	41869	0.5	93	48792	1.91	3.87	(2.41, 6.22)***	<0.001	3.92	(2.43, 6.33)***	<0.001
AGis	.	.	.	.	.	.						
No	20576	4434815	4.64	44504	4031348	11.04	2.37	(2.33, 2.41)***	<0.001	2.31	(2.27, 2.35)***	<0.001
Yes	153	64701	2.36	739	70695	10.45	4.42	(3.71, 5.26)***	<0.001	4.19	(3.51, 4.99)***	<0.001
GLP1RAs												
No	<3	NA	4.61	<3	NA	11.03	2.38	(2.34, 2.42)***	<0.001	2.33	(2.29, 2.37)***	<0.001
Yes	<3	NA	1.3	<3	NA	2.03	1.54	(0.3, 7.94)	0.6073	10.46	(1.01, 108.29)*	0.0489
Insulin	.	.	.	.	.	.						
No	20023	3896115	5.14	43215	3698679	11.68	2.26	(2.22, 2.29)***	<0.001	2.26	(2.22, 2.3)***	<0.001
Yes	706	603401	1.17	2028	403364	5.03	4.24	(3.9, 4.62)***	<0.001	4.37	(4.01, 4.77)***	<0.001
Number of AHMs												
0	18675	3511461	5.32	35071	3042989	11.53	2.16	(2.12, 2.2)***	<0.001	2.08	(2.04, 2.12)***	<0.001
1	1705	714993	2.38	8312	783794	10.60	4.39	(4.17, 4.63)***	<0.001	4.17	(3.95, 4.39)***	<0.001
≥2	349	273062	1.28	1860	275260	6.76	5.30	(4.73, 5.94)***	<0.001	5.56	(4.96, 6.24)***	<0.001

AHMs, anti-hyperglycemic medications; aHR, adjusted hazard ratio; AGis, alpha-glucosidase inhibitors; AS, ankylosing spondylitis; cHR, crude hazard ratio; CLD, chronic liver disease; CKD, chronic kidney disease; DM; diabetes mellitus; DPP4is, dipeptidyl peptidase-4 inhibitors; GLP1RAs, glucagon-like peptide-1 receptor agonists; IR, incidence rate, per 1,000 person-year; LDH, lumbar disc herniation; MM, multiple myeloma; N, number of events; NA, not applicable; PY, person-year; SGLT2is, sodium–glucose cotransporter 2 inhibitors; SUs, sulfonylureas; TZDs, thiazolidinediones; 95% Cl, 95% confidence interval.

† Adjusted hazard ratio estimated by multivariable analysis including sex, age, comorbidities, and anti-hyperglycemic medications.

*P <0.05, **P <0.01, ***P <0.001.

In accordance with the guidelines of the National Health Insurance Research Database for safeguarding patient privacy, the data retrieval from the database is not possible as the event count is less than three. Therefore, it will be indicated as "<3" to ensure confidentiality.
